# Analysis of Artisanal Small-scale Gold Mining Sector in West Sumbawa Regency, Indonesia

**DOI:** 10.5696/2156-9614-6.12.26

**Published:** 2016-12-19

**Authors:** Baiq Dewi Krisnayanti, Ivano Vassura, Maywin Dwi Asmara, Ardiana Ekawanti, Herman Suheri

**Affiliations:** 1 Independent Researcher; 2 Department of Industrial Chemistry, University of Bologna, Italy; 3 Graduate Student, Agriculture Faculty, University of Mataram, Indonesia; 4 Medical Science Faculty, University of Mataram, Indonesia; 5 Agriculture Faculty, University of Mataram, Indonesia

**Keywords:** ASGM, West Sumbawa, mercury

## Abstract

**Background.:**

The high value of gold reserves in West Sumbawa Regency (WSR) and West Nusa Tenggara Province, Indonesia has resulted in an increase in small-scale gold mining activity in this area. Artisanal and small-scale gold mining (ASGM) is an attractive alternative livelihood for rural workers because it has good potential to improve the wealth of a community. Miners need very little training to mine gold and the transition from traditional farming or fishing is easy to make. However, the key environmental consequence of ASGM in West Sumbawa is the extensive use of mercury and its impact on human health.

**Objectives.:**

The ASGM activity in WSR is quite recent when compared to other ASGM activity in Indonesia. The current study was conducted to better understand the lifestyle, extent of mercury exposure, and the health of people living in WSR, West Nusa Tenggara Province, Indonesia.

**Methods.:**

The present study was designed as a purposive field sampling study conducted in WSR, West Nusa Tenggara Province, Indonesia. The subjects were miners and families from three different sites within the WSR: individuals directly exposed to mercury, indirectly exposed individuals and non-exposed individuals. Hair mercury analysis was done with all subjects. Health questionnaires, physical examinations and socio-economic surveys were conducted with exposed subjects.

**Results.:**

The ASGM sector in the WSR consists of a high number of migrant workers who have a great economic impact on the local area, high mercury use, a great deal of illegal mercury trading, and a high mercury concentration (>13 mg/kg) in their hair. The results suggest that ASGM activities affect the health of exposed and indirectly exposed individuals.

**Conclusions.:**

The current scale of ASGM activity in the WSR is predicted to rise. ASGM activities in the WSR is an important challenge that needs to be addressed.

## Introduction

Mercury contamination is a critical issue for human health. Although mercury is naturally present in the earth, human activities have expanded its presence.^1^ A global action to protect human health and the environment from anthropogenic emissions and releases of mercury and mercury compounds was signed in October 2013 under the Minamata Convention of the United Nations Environment Programme (http://www.mercuryconvention.org). Mercury is typically used in artisanal and small-scale gold mining (ASGM) activities. Even though many mercury-free methods have been introduced to miners in developing countries around the world, mercury is still a common method used to recover gold. ASGM activities contribute to around 25% of worldwide gold production and 38% of worldwide mercury emissions yearly.^2^,^3^

Other well-reported mercury exposure pathways include ingestion of mercury through diet, particularly fish.^4^,^5^ Rice in inland areas is also now considered to be a major vector for mercury ingestion.^6^,^7^

ASGM has been practiced throughout Indonesia for many years. There are many reports of operations in Kalimantan (Palangkarya, Banjarmasin), Sulawesi (Manado, Palu) and Java (Pongkor, Tasikmalaya), with the most recent mining developments on the island of Sumbawa. The West Sumbawa Regency (WSR) is a meeting place of two tectonic plates, the Indo-Australia (south) and Eurasian (north) plates.^8^ The associated geological conditions have endowed Sumbawa Island with significant mineral resources including gold (180,000 m^3^), encouraging mining activity in West Sumbawa.^9^

Artisanal and small-scale gold mining is an attractive alternative livelihood for rural workers because of its potential to improve the wealth of a community. Miners need very little training to mine gold and the transition from traditional farming or fishing lifestyles is easy to make. In addition, miners often travel from one mine to another. ASGM work involves digging, grinding, and crushing the ore, then processing the ore, ending with burning of the amalgam to attain the gold. Most miners are men. Few women work as miners, and mainly as ore crushers, not as smelters. ASGM activity in the WSR is relatively recent compared to other ASGM activity in Indonesia. Describing the ASGM sector in WSR and its impact on environment, health and society is necessary for developing a better solution to eliminate mercury use in the ASGM sector. The current study was conducted to better understand the lifestyle, extent of mercury exposure, and health of people living in the ASGM mining areas of West Sumbawa Regency, West Nusa Tenggara Province, Indonesia.

**Abbreviations**
*ASGM*: Artisanal small-scale gold mining
*PPE*: Personal protective equipment
*WSR*: West Sumbawa Regency

## Methods

### Study Location

**Figure 1:**
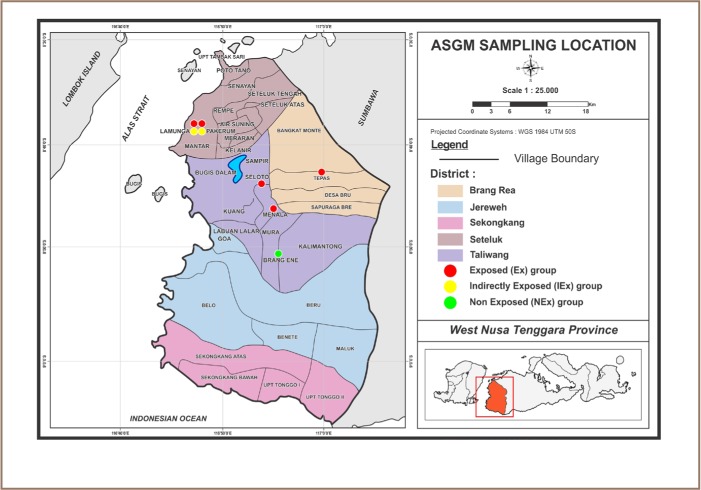
Sampling locations in West Sumbawa Regency of West Nusa Tenggara Province, Indonesia

Figure 1 shows the West Sumbawa Regency, and West Nusa Tenggara Province, Indonesia. A total of 150 subjects were recruited from three different groups: exposed individuals, indirectly exposed individuals, and non-exposed individuals.

- Exposed subjects were miners (cylinder operators, rock diggers, smelters) and also miners' families (wives and children) who live in the gold mining processing areas of Tepas, Menala, Seloto, Lamunga and Pakerum villages. Miners usually bring their family to live close to their place of employment. Ninety exposed subjects were recruited.
- The indirectly exposed group was comprised of individuals who lived within 0.5 km of the ASGM site, but who were not involved in ASGM activities on a daily basis. These subjects were recruited from 2 villages only (Pakerum and Lamunga). Thirty indirectly exposed subjects were recruited.
- The non-exposed subjects were recruited from Brang Ene district, about 5 km from an ASGM site. There are no ASGM activities upstream from this village. Thirty non-exposed subjects were recruited.

### Hair Sampling

A human subjects research permit was granted by the Ethics Committee of the Medical Faculty of the University of Mataram, Indonesia. Informed consent was obtained from all subjects at the time of enrollment. Hair samples were collected from all 150 subjects. The 150 hair samples were collected according to the method described by the United Nations Environment Programme, where approximately 150 mg of hair was cut from the scalp of the occipital region with clean stainless steel scissors, then sealed in labeled plastic bags.^10^

Hair mercury analysis was carried out at the Industrial Chemistry Department of Bologna University, Italy. Prior to analysis, hair was washed to remove surface contamination following the procedure recommended by the International Atomic Energy Agency, where hair samples were first rinsed with ultrapure water, then washed with acetone three times, followed by a final wash three times with ultrapure water.^11^ The washed hair was dried in an electric oven at 60°C until the weight was stabilized (overnight), and cut into small pieces (less than 4 mm) with stainless steel scissors. Approximately 120 mg of dry hair from each sample was added to a 100 ml Teflon acid digestion vessel, and 2.5 ml concentrated nitric acid was added. The digestion vessel was closed and then digested in a microwave oven at 180°C for 120 minutes (Anton Paar 3000). The digest solution was then diluted to 15 ml with ultra pure water, and brought to a final volume of 50 mL with 5% hydrochloric acid. The total mercury concentration in solution was quantified using atomic absorption spectroscopy (Perkin Elmer AAnalyst 400) equipped with a MHS-15 mercury hydride system. A NaBH^4^ mercury hollow cathode lamp was used as the radiation source. Sample blanks, standard blanks and analytical duplicates were used to ensure the accuracy and precision of the analysis. A Perkin Elmer Analyst 700 (Norwalk, CT, USA) atomic absorption spectrometer was used in the present study. All measurements were carried out using high purity argon. A mercury analytical lamp was used with 220V, C-EDL lamp type, 253.65 wave length and slit 2.7/1.05. For the reducing agent, sodium borohydride (3% w/v) in sodium hydroxide (1% w/v) was used. The detection limit of mercury was 0.008 μg/L. In addition, a precision of 0.95% relative standard deviation was acquired at the level of 1.2 μg/L, and the correlation coefficient of standard curve equation in each test reached at least 0.999.

### Health Questionnaire and Physical Examination

Following hair collection, members of the exposed subgroup were asked to participate in a health assessment questionnaire, and follow up physical examination. Fifty-five out of the 90 subjects in the exposed subgroup were willing to undergo the physical examination and respond to the health questionnaire. The physical examination assessed subjects for symptoms of mercury poisoning, including bluish discoloration, ataxia of gait, dysdiadochokinesia, and finger to nose tremor. The neuro-psychological test also included matchbox tests for signs of coordination problems, ataxia and tremors, and pencil tapping tests, which assess the presence of coordination problems and tremor.^10^ The health questionnaire collected information to better understand the medical history of subjects before and after exposure to mercury.

### Socio-Economic Survey

A socio-economic survey was conducted among the same 55 subjects from the exposed group that agreed to the health questionnaire and physical examination. The socio-economic survey collected information on the nature of mining employment in WSR, including questions on the economic status of subjects, reasons for participating in ASGM, income, personal protective equipment (PPE) use, amount of mercury use, previous employment, and job description.

## Results

### Mercury Concentrations in Hair

Table 1 shows that the exposed subgroup was composed of 17 females and 73 males (81%), and the mean age of exposed subjects was 35 years. In the exposed subgroup, 14 people were not directly engaged in mining extraction activities: 9 women, 2 men and 3 children (5, 9 and 12 years old). The indirectly exposed and non-exposed subgroups did not show a statistically different age distribution but the percentage of males was lower than in the exposed subgroup.

**Table 1:** Descriptive Statistics of the Three Population Subgroups

***Subgroup***	**Age (years)**							

	**Size**	**Male (%)**	**Max**	**Min**	**Mean**	**Median**	**25%**	**75%**
**Exposed**	90	81	71	5	35.0	33.5	24	44
**Indirectly exposed**	30	43	65	7	33.9	33.5	20	45
**Non exposed**	30	47	59	9	30.4	28.5	20	42

**Figure 2:**
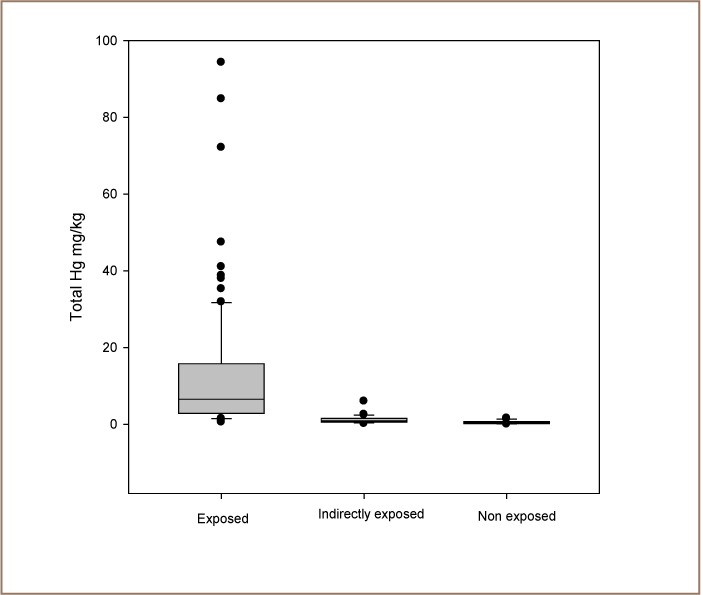
Distribution of total mercury concentration in head hair samples from the three subgroups

It can be seen from Figure 2 that the exposed subgroup had very high levels of total mercury in their hair compared to the indirectly exposed and non-exposed subgroups. The total mercury concentration in the exposed subgroup was above the alert level of the human biomonitoring threshold level.^12^

The total mercury level in the indirectly exposed subgroup was also high, but the total mercury level in the non-exposed subgroup was below the World Health Organization permitted level.

The median concentration of total mercury detected in hair collected from non-exposed subjects was 0.45 mg/kg (*Table 2*). This value was about 14 times lower than the mean concentration detected in hair collected from the exposed group. Evidence of mercury exposure was found in the indirectly exposed subgroup. Furthermore, high levels of mercury were also found in exposed children (mean 4.7 mg/kg, median 5.0 mg/kg) (*Table 3*) and in exposed subjects living in the mining areas, but who were not engaged in mining activities (mean 5.6 mg/kg, median 5.0 mg/kg). Among vulnerable groups (women and children), levels reached 16 mg/kg for women and 4.7 mg/kg for children. The median and mean hair mercury levels in the indirectly exposed subgroup were twice those of the non-exposed subgroup. The Mann-Whitney U test confirmed that the difference in the median values of the two groups was greater than would be expected by chance, therefore there was a statistically significant difference between the subgroups (P = <0.001).

**Table 2:** Mercury Concentrations in Hair Samples by Subgroups

***Subgroup***	**Size**	**Max mg/kg**	**Min mg/kg**	**Mean mg/kg**	**Median mg/kg**	**25%**	**75%**	**Acceptable level (mg/kg)^10^**
**Exposed**	90	94	0.56	13	6.5	2.9	16	1
**Indirectly exposed**	30	6.0	0.19	1.3	0.90	0.56	1.5	
**Non exposed**	30	1.6	0.05	0.56	0.45	0.23	0.74	

**Table 3:** Total Mercury Concentrations by Age and Gender

	**Exposed**			**Indirectly Exposed**			**Non-Exposed**		
		
	**Child (<13 years old)**	**Adult Female**	**Adult Male**	**Child (<13 years old)**	**Adult Female**	**Adult Male**	**Child (<13 years old)**	**Adult Female**	**Adult Male**
**Sample Size**	3	15	72	3	15	12	4	13	3
**Mean (mg/kg)**	4.7	16	13	1.5	0.9	1.7	0.4	0.48	4.7
**Median (mg/kg)**	5.0	6.6	6.5	1.5	0.7	1.4	0.35	0.40	5.0

### Health Questionnaire and Physical Examination

According to the results of the health questionnaire, 22% of the 55 respondents smoked 24–36 cigarettes/day, 49% smoked 3–12 cigarettes/day, and 29% were non-smokers. The hair results showed that the total mercury levels of smokers were relatively higher (2–72 mg/kg) than that of non-smokers (2–10 mg/kg). Statistical significance was not tested. Similar studies have indicated that smoking habits influence the total mercury concentration in the human body, with total mercury levels being higher in the body of smokers body compared to non-smokers.^13–16^

29% of respondents reported consuming sea food or fresh water fish every day, 43% consumed fish 3–4 days/week, 21.5% consumed fish 1–2 days/week, and 6.5% reported that they did not eat fish. None of the respondents knew where the sea food that they consumed came from, as they purchased it in local markets or from door to door merchants. For the fresh water fish, respondents reported that the fish came from the nearby river and lake. Many ASGM spots are located uphill from a lake or river, therefore there is a chance that mercury-containing mine tailings may contaminate these water ways through runoff or infiltration. However, there were no significant differences (p=0.07) in potential co-founders such as smoking or fish consumption on mercury intoxication.

Table 4 shows the symptoms most commonly experienced by the members of the exposed group that consented to the health questionnaire and physical examination were finger tremors and sleep disturbances. In addition, respondents reported frequent excessive salivation, physical fatigue and on the neuro-psychological tests (matchbox and pencil tapping tests), respondents had a high frequency of positive results.

**Table 4:** Results of Health Questionnaire and Physical Examination

***Examination (n = 55)***	**Symptoms**	**Number with positive result**
**Questionnaire data**	Metallic taste	10
	Excessive salivation	12
	Tremor fingers	27
	Sleep disturbances	15
	Regular flu	2
	Severe fatigue	1
	Severe headache	1
**Physical examination**	Disc (bluish discoloration)	23
	Ataxia of gait	5
	Dysdiadochokinesia	21
	Finger to nose tremor	5
	Matchbox test	
	0	15
	1	0
	2	40
	Pencil tapping test	
	0	6
	1	12
	2	37

Note: neuropsychological data: 0 = good performance 1 = restricted performance 2 = bad performance

Table 5 shows that of the 55 exposed subjects that completed the questionnaire, 62% had hair total mercury concentrations higher than the human biomonitoring threshold level of 5 mg/kg.^12^ Diagnosis of chronic intoxication was defined when human biomonitoring was greater than the threshold and the physical examination score was a minimum score of 5. As a result, 44% of participants showed chronic mercury intoxication, while the remaining 56% showed no mercury intoxication. Several subjects with a low level of hair mercury demonstrated a high score, and some with a high hair mercury level and a low score did not show mercury intoxication.

**Table 5:** Summary of Human Biomonitoring (Hair Mercury) and Physical Examination Scores for all Subjects

***Medical result***	**Median; mg/kg (25%–75%)**	**Percentage**
**Hair Hg (mg/kg)**	6.4 (2.9–21)	
**Hair Hg < HBM**	2.5 (1.7–3.6)	38% (n=19)
**Hair Hg > HBM**	16.4 (6.6–27)	62% (n=31)
***Medical score***		
**0–4**		34.5% (n=19)
**5–9**		54.6% (n=30)
**10–19**		10.9% (n=6)
***Summary Hair Hg and score***		
**Hair Hg < HBM**		
		
**0–4**		36.8% (n=7)
**5–9**		52.7% (n=10)
**10–19**		10.5 %(n=2)
**Hair Hg > HBM**		
**0–4**		29.0% (n=9)
**5–9**		58.1% (n=18)
**10–19**		12.9% (n=4)

Abbreviations: Hg, mercury; HBM, human biomonitoring

### Socio-Economic Status

The socio-economic survey found that 47% of the respondents came from Lombok Island, 43% of the respondents were local people from around Sumbawa Island, and 10% of the respondents come from another island (Sulawesi, Kalimantan and Java Islands). Most of the cylinder owners and operators in WSR came from Lombok Island. This accounts for migrant workers directly involved with mining: crushing, grinding and processing the gold ore. This figure does not account for individuals indirectly involved with the ASGM sector through the provision of support services and infrastructure (e.g. food, transportation, security, housing, etc).

A comparison of miners' incomes before and after working in ASGM showed that ASGM activities have resulted in an increase in community income. The average income of miners prior to working in ASGM was € 2,05/day or less, and after switching to mining, the average income was € 30.08/day or more (*Table 6*). Migrant workers' stated reasons for coming to WSR was the greater opportunity for increased income compared to their previous residence (80%) and unemployment (20%). Most of the migrant workers from Lombok Island had been involved in ASGM since 2010, 10% had a mining background from Kalimantan (previously worked in Kalimantan, came back to Lombok, then moved to Sumbawa Island for mining). Miners' had previously worked as farmers, casual workers, graduate students, merchants, drivers, etc.

**Table 6:** Average Income Before and After Involvement in ASGM by Village

***Village***	**Average income prior to mining(€ per day)**	**Average income of miners(€ per day)**
**Seloto**	2.85	43
**Tepas**	1,53	26.06
**Menala**	1,47	7,7
**Pakerum**	2,31	37,23
**Lamunga**	2.11	40.11

Mining activity using the amalgamation system (cylinder) generally begins at 8 am with a workday of 10–12 hours, 5–7 days per week. Most miners have no formal training and learn their jobs from friends and watching other miners. Furthermore, all miners agreed that mining is a dangerous, unsafe, insecure and difficult occupation. However, they also felt that mining employment was necessary to feed their family, and preferable to no occupation, or resorting to criminal activity.

Information was obtained about the extent to which miners used PPE to reduce the risk of direct inhalation or direct contact with mercury. Around 20% of the WSR miners knew about PPE and used masks during the burning of amalgam, 33% of the miners knew about PPE, but did not use masks, gloves or boots, and 47% of the miners said that they were not aware of PPE. However, direct mercury exposure is not limited to amalgam burning, as most miners used their bare hands to squeeze amalgam through cloth to separate excess mercury.

We did not count the number of cylinders in WSR to determine the amount of mercury used in WSR, but rather used the number of amalgamation cylinders in WSR which was 5000 cylinders.^17^ According to the survey, miners use an average of 250–500 grams of mercury/cylinder. It was therefore estimated that the amount of mercury use in WSR was more than 1.25 tons per day. To determine the amount of mercury distribution in the WSR, from the questionnaire, it was calculated that every month 24% of the miners buy 4 kg of mercury, 3% buy 3 kg mercury/month, 54% buy 2 kg mercury/month, and 19% buy 1 kg mercury/month. This means that (per 5000 cylinders) the miners use around 384,15 kg mercury/day. The price of mercury in 2014 was 100 euro per kg, indicating that the mercury business in WSR was 38,400 euro/day.

## Discussion

West Sumbawa's ASGM activities continue to grow due to the continued market for gold and the low underlying capital equipment requirements and operational expenses. ASGM activities in West Sumbawa can produce positive monetary benefits for miners and expand local economies. As a consequence, the population of West Sumbawa has increased over the last 5 years by 3.5%, expanding local economies through support services and infrastructure, such as food, transportation, security, and housing. Prior to becoming involved in mining activities, miners earned €2.05/day or less. With the increasing income from ASGM activity, miners can increase their quality of life. However, detrimental health effects have been found as a result of ASGM, as miners who were exposed to mercury an average of 6 years showed a high level of mercury intoxication. The brief exposure time demonstrates that the clinical findings reflect a high dose mercury used in this area and that the route of exposure was by mercury inhalation, which results in higher exposures than food intake. Mercury residue was not only found in miners who were directly exposed to mercury on a daily basis, but also in family members who were only indirectly involved in ASGM activities. Attention also should be focused on the indirectly exposed subgroups (housewives, farmers and casual workers).

Due to concern over the effect of mercury on human health, many efforts have been made by non-governmental organizations and other institutions to introduce mercury-free methods for recovering gold in the WSR, such as using borax or panning. Unfortunately, these alternative methods have not yet been adopted by WSR miners, for a variety of reasons: different types of gold ore, impracticality, increased time consumption, and high water usage, etc. It has been suggested that the cyanidation system is the best way to reduce mercury use, and this method is highly recommended as the cylinder is only used for grinding the rock for further processing by the cyanidation system, without adding mercury to the cylinder. However, there is no guarantee that miners will adopt these methods, as there are no regulations imposing alternative methods. Airborne mercury emission is detrimental to human health, even to those not directly included in gold mining activities. Furthermore, there is no framework for filtration of mercury vapors in order to burn amalgam to protect human health. There have many attempts to address this issue, but they have not been easily adopted. Thus, the burning of amalgam must be prohibited in urban areas. However, the burning of amalgam is practiced by miners in open spaces and this is difficult to prevent without regulatory intervention. Miners are currently free to use whatever methods they choose without regulation, including the high use of mercury. Therefore, it is necessary to improve our understanding of the current mining practices across ASGM sites, including the motivations and interests, as well as barriers to miners in order to facilitate the introduction of appropriate and effective interventions.

It has been proposed that the best way to ensure positive results on health, safety and the environment with regard to ASGM activities is formalization of the ASGM movement. However, formalization of this sector remains a huge challenge. The absence of formalization in ASGM broadly hampers the ability of miners to actualize changes, specifically the absence of access to formal credit markets as a result of the informal (and sometimes illegal) nature of this sector, and is also an obstacle to implementing changes. Miners require access to capital to implement changes that permit the utilization of alternative mining practices, and formalization would promote positive financial conditions for miners and local communities. In 2012, the government of WSR issued a decree aimed at stopping ASGM activity, but it failed, and ASGM activity persists and is growing. In 2014, the government worked together with PT Indotan, a mining concession holder in the WSR, to formalize ASGM activities. This formalization began by inviting miners to become members of a cooperative. Cooperative formalization would help to integrate the social, environmental, labor, health and safety, economic, commercial, gender, organizational and technical dimensions of AGSM activities. However, these processes require time and a consistent long-term governmental policy. With formalization, the government would have the power to impose interventions with legal consequences for both sides (government and miners). In spite of formalization, it is suggested that regular monitoring of human health of all subgroups is necessary in order to control current methods and receive the most appropriate treatment and care. The increased cost of safer extraction methods such as cyanidation could be offset by the increased efficiency of cyanide versus that of mercury in recovering gold.^18^

One important limitation of the present study is the possibility of selection bias. Sample bias may have occurred if only miners experiencing symptoms of mercury toxicity agreed to the physical examination, and thus the assessment of the exposed subgroup may not be generalized to those who were not examined. Selection bias is less likely in the indirectly and non-exposed subgroups. As a result, the differences between these two sub-groups and the exposed group may have been overestimated. Nevertheless, the medical symptoms and biometric measures were consistent with high dose mercury exposure and provide an indication of the danger of mercury amalgamation.

## Conclusions

The ASGM sector in the WSR has a large migrant worker population, which is an important economic support to the local community, but ASGM activities involve high mercury use and illegal mercury trading. These activities have affected the health of miners in a short time, as evidenced by the high mercury residue on the bodies of miners. Formalization of ASGM in the WSR is a important challenge that needs to be met.
